# Oligometastases in prostate cancer: restaging stage IV cancers and new radiotherapy options

**DOI:** 10.1186/s13014-014-0258-7

**Published:** 2014-12-11

**Authors:** Antonio José Conde Moreno, Carlos Ferrer Albiach, Rodrigo Muelas Soria, Verónica González Vidal, Raquel García Gómez, María Albert Antequera

**Affiliations:** Servicio de Oncología Radioterápica, Instituto Oncológico de Castellón “Dr. Altava”, Consorcio Hospitalario Provincial de Castellón, Av. Dr. Clarà N 19, 12002 Castellón de la Plana, Castellón Spain

**Keywords:** SBRT, SRS, Prostate metastases

## Abstract

There are various subgroups of patients with metastatic prostate cancer: polymetastatic, oligometastatic, or oligo-recurrent cancers whose progression follows different courses and for whom there are different treatment options. Knowledge of tumor dissemination pathways and different genetic and epigenetic tumor profiles, as well as their evolution during disease progression, along with new diagnostic and therapeutic advances has allowed us to address these situations with local ablative treatments such as stereotactic body radiation therapy or stereotactic radiosurgery. These treatments provide high rates of local control with low toxicity in metastatic spread for primary cancers including those of pulmonary, digestive, and renal origin, while these types of treatments are still emerging for cancers of prostatic origin. There are several retrospective studies showing the effectiveness of such treatments in prostate cancer metastases, which has led to the emergence of prospective studies on the issue and even some phase II studies intended to prevent or delay systemic treatments such as chemotherapy. Here we collect together and review these past experiences and the studies currently underway. These types of radiotherapy treatments redefine how we approach extracranial metastatic disease and open up new possibilities for combination therapy with new systemic treatment agents.

## Introduction

### Restaging stage IV cancer

Beginning with Halsted [1] in 1907 various theories for tumor dissemination have been proposed. The first important theory involved the locoregional lymphatic pathway and hypothesized that the disease could be cured if diagnosed at an early stage and if aggressively surgically managed. More than 70 years later, a second model became popular in oncological practice: using the breast cancer model (also used by Halsted), this model proposed that cancer is a systemic disease that always metastasizes and thus will already have done so early in the disease course, meaning that local therapies are less important than the tumor microenvironment or systemic therapies [2-4]. Later a third theory was proposed based in the "Spectrum hypothesis" [5] according to which the disease ranges between local and disseminated at the time of diagnosis. However, none of these theories has been tested in randomized clinical trials at the biological level [6].

Progression describes the cause of cancer as the accumulation of acquired somatic mutations and chromosomal rearrangements which gradually add up over a long period of time, progressively converting normal epithelium into neoplastic cells which later acquire a metastatic phenotype [7]. According to the "seed and soil" theory once cells acquire this phenotype they gain a lymphogenic and angiogenic capacity for intravasation, adhesion, and an ability to cause micrometastases [8] which can thrive in any organ [9]. Certain tumors are not sufficiently robust in one or more of these steps, referred to as “tumor dormancy”, which prevents the cancer from otherwise progressing. The patient’s immune system appears to play a key role in this dormancy [10]: This system has the ability to kill tumor cells, but there may be balance situations in which it cannot completely eliminate the disease and thus the cancer remains dormant until an immunoediting phenomenon occurs which prevents these cells from being perceived as antigenic by the immune system, thereby allowing the disease to develop [11].

However, phenomena such as tumor mutations, adaptations, and changes in their genetic and epigenetic profiles cannot be explained by the theory of progression and so new tumor models are emerging. Based on data uncovered by the human genome project, Stephens et al. [12] proposed the “chromothripsis theory” whereby hundreds of genomic adjustments occur in an isolated cell event which would be unlikely to occur in an aggregated and a random manner accumulating over time; In this theory key mutations among these adjustments trigger catastrophic molecular changes which cause cancer. This phenomenon has been observed in at least 2-3% of all tumor sub-types and there is evidence for its involvement in more than 25% of bone tumors. According to this theory near catastrophic chromosomal breakage occurs followed by telomere failure or dysfunction causing the union of different non-homologous genes which could lead to the loss of tumor suppressor genes and/or the activation of oncogenes. Phenomena with different patterns of DNA breakage and hypermutability have also been described in the prostate cancer model [13] suggesting that a limited number of metastases (oligometastases [6]) may occur early on which later seed others after they become genetically unstable.

If metastases arise from clonal expansion [14] clones with selective advantages would give rise to them, therefore destroying these clones (for example with ablative radiotherapy techniques) would decrease their metastatic potential. Consequently it is very important to distinguish between different oncological situations; oligometastases, term introduced by Weischelbaum and Hellman, describes a situation where a patient has distant disease in a limited number of regions (less than five) and where the primary lesion can be controlled [5]. Yazuro Niibe [15] described the term oligo-recurrence where a patient presents one or more distant metastases or recurrences in one or more organs, and in addition to the primary tumor being under control, these metastases or recurrences may be amenable to local treatment, leaving the possibility that no future disease additional to that already described would occur. Niiebe, Onishi, Chang and other experts [16] published a prognostic classification based on the location and number of metastases, histology, and the moment of their presentation along the course of the disease. This classification specifically focusses on lung cancer but other histologies could be included.

Another important method of metastasis classification is Rubin’s amended TNM staging system [17]. He suggests modifying the "M" to represent solitary metastasis (M1), oligometastases (M2), or multiple metastases (M3), and adding an "S" (depending on the presence and levels of any serological markers), an "H" (using a modified Karnofsky scale to determine the condition of the patient), and finally an "A" or "B" based on whether the patient is symptomatic or not. The first two factors are important for deciding upon and individualizing treatments and the latter two take the patient’s general overall condition, which is a better predictor of outcome, into account rather than age specifically.

### Prostate cancer metastases

#### Patterns of prostate cancer dissemination

Bubendorf et al. [18], in a series of 19,316 autopsies, of which 1,589 presented prostate cancer, metastases were detected in 35%: 90% were bone metastases, followed by lung (46%), liver (25%), pleura (21%), and adrenal metastases (13%); 90% of the bone metastases involved the spinal cord. In terms of the pathways or patterns for this spread, there is an eminent pattern of local extension which occurs through the capsule, seminal vesicles, and bladder, although Apex tumors may have an increased extracapsular extension capacity due to their small capsule size. The Denonvilliers fascia creates a natural barrier to the rectum so extension at this level is rare. Another important pathway is the lymphatic system, which is involved in 9 to 16% of cases, especially at the level of obturator, presacral, internal, and common iliac arteries, and sometimes even the extra-regional paraaortic chains. Hematogenous dissemination can also occur through the cava to the liver and lungs.

Venous retrograde invasion through the Batson plexus, leading from the prostate to the periprostatic veins and presacral veins, column, and subsequently to the lungs is one of the main routes of dissemination in metastatic patients. Indeed, there is evidence which proves this mechanism, such as the inverse relationship between spinal and pulmonary metastases. Spinal cord involvement most often occurs in smaller tumors (4 to 6 cm), while lung involvement is more likely in large tumors (6 to 8 cm), with liver involvement usually restricted to the largest tumors (8 cm or larger), suggesting that spinal metastases precede lung and liver metastases. Further supporting this idea, a gradual decline in lumbar spinal and cervical vertebrae involvement (97% to 38%) [19,20].

#### Diagnosis of metastasis in prostate cancer

The role of PSA (prostatic specific antigen) should be highlighted in prostate cancer: it is a highly predictive marker for bone metastases, with a positive predictive value of 74% when present at a concentration greater than 100 ng/mL and a negative predictive value of 98% when present at less than 10 ng/mL. Other serological tests, such as models based on circulating tumor cells, are still in development.

CT (computed tomography) plays an important role in detecting bone (mainly blastic), lung, and liver metastases and MRI (magnetic resonance imaging) is useful in the diagnosis of liver, and especially, spinal metastases. New non-parametric techniques such as whole-body (WB) MRI and diffusion-weighted imaging (DWI) studies have proven useful for detecting lymph node, bone, and visceral metastases [21-26].

Undoubtedly, modern techniques such as PET (positron emission tomography) and CT have the greatest versatility in terms of metastasis detection. Different radiolabels that are useful for prostate cancer diagnosis and tracking are in development, including markers of cell membrane proliferation (11C/18F-choline), fatty acid synthesis (11C- acetate), amino-acid transport and protein synthesis (11C- methionine), androgen receptor expression (18F- FDHT), sodium fluoride labeled with fluorine 18 (sodium fluoride F 18 [18F-NaF]) and osteoblast activity (18F-fluoride). In the case of the latter, there is more reported experience with 11C-choline and 18F-fluorocholine [27]; the main difference between two is their half-life, which is considerably longer for 18F-fluorocholine compared to 11C-choline (20 min vs. 110 min respectively) [28]. In addition, recently studies stand out showing the role of 18F-NaF PET/CT as a highly sensitive modality in revealing sites of occult osseous metastases in prostate cancer [29,30] , moreover growing evidence suggests that provides also increased specifity in the detection of bone metastases and that combined with other tracers such as F-FDG could provide additional information [31-33].

Since the discovery of PET/CT, many authors have proposed the use of these techniques in the diagnosis and staging of prostate cancer and for restaging local and systemic relapse patients, albeit with mixed results. In order to try to unify these varied criteria and conclusions Umbehr et al. [34] published a meta-analysis in collaboration with the European Urology Association which included 44 studies with 2293 patients, 25 of them were in a staging setting and 19 were in relapse (restaging setting). In patients for whom PET-CT was used for initial staging, a sensitivity of 84% and specificity of 79% was reached; the results were better for restaged patients where a sensitivity of 85% and specificity of 41.4% was obtained. However, the authors assumed some limitations, such as heterogeneity among the variables included in the different studies, which prevented them from stratifying the sample. They concluded that although the use of PET-CT allows the correct staging and restaging of patients with prostate cancer who have received prior radical treatment, it should not be considered as a gold standard test in all patients. Correct patient selection is necessary in order to avoid false negatives.

Thus, when using this method in staging, high-risk patients with a Gleason score of 8–10 and elevated PSA levels (20 ng/ml or more) are the groups most suitable for radical treatment [35]. In contrast, the most important predictive variables for patients in the restaging group correspond to low levels of PSA (1 ng/ml or more), short cell doubling times (less than 3 months and up to 6), and an initial staging higher than pT3b or pN1 [36,37]. Therefore, despite all these advances we still lack an accurate, direct, and reproducible “gold standard” imaging technique to delineate metastasized prostate cancer.

#### Selection criteria for ablative therapy in metastatic prostate cancer

Current guidelines, such as those from the National Comprehensive Cancer Network (NCCN) [38] do not include the possibility of ablative treatment for metastatic prostate cancer, relegating the role of radiotherapy to that of a palliative treatment for symptomatic patients. This is in contrast to other pathologies, for example, colorectal and pulmonary tumors where radiotherapy already has a clear role [39,40]. The origin of this indication is based on the good results obtained in a series of liver and lung metastasectomies, although this does not exclude the possibility that there are some oligometastatic prostate cancer patients who could also benefit from such local treatments [41,42].

In 2004 the University of Rochester [43] studied which patient subgroups are most likely to develop oligometastases. Using retrospective data from 369 patients who were treated with external radiotherapy for primary prostate lesions, the course of 74 patients (20%) who developed metastases was studied: A follow-up over 10 years later determined that there was a significant increase in overall survival (OS) in bone metastasis cases (58% at 5 years and 27% at 10 years). They also noted that pelvic bone metastases were associated with reduced survival when compared to spinal metastases. In fact, patients with less than five metastases had better survival compared to those with more than five metastases (73% and 36% vs. 45% and 18% at 5 and 10 years respectively). The metastasis-free interval after the prostate cancer diagnosis was higher in those with less than five lesions compared to those with more. It was also found that survival from the time of the diagnosis of bone metastases to death was not statistically significant between these groups. Thus it was concluded that early detection and aggressive treatment with surgery or radiation is the best course of action.

Piet Ost et al. [44] tried to identify what prognostic factors influence prostate cancer-specific survival (PCSS) in non-castrated patients with metastatic prostate cancer, retrospectively analyzing 80 such patients. Univariate analysis of the time interval from diagnosis to metastasis, the pre-metastatic PSA duplication time (PSA-DT), the number of metastatic lesions, pattern of metastatic spread (especially lymph node and bone involvement), and the treatment received at the time of metastases detection were significant predictors for PCSS. However, only pre-metastatic PSA-DT, the pattern of metastatic spread, and the number of metastases were significant predictors in the multivariate analysis. The number of metastases found retained their ability to predict PCSS even to a cutoff of one lesion, but when the cutoff was between three and five they were not a significant predictor in multivariate analysis. Trying to identify a poor-prognosis subgroup, they stratified patients according to the number of metastasis (less than one metastasis to more than one), and PSA-DT (three months or less to more than three). All patients with one metastasis and a PSA-DT of more than three months were alive at five years compared to only 8% of patients more than three and a PSA-DT of less than three months. The authors concluded that a subgroup of patients with a longer PSA-DT, node or axial skeleton involvement, and a lower number of metastases have a better PCSS in non castrate patients developing metastases.

According to present knowledge, the criteria for performing ablative treatment in metastatic patients, and particularly metastatic prostate cancer, patients would be: oligo-recurrent patients (with a controlled or controllable primary lesion) with five or fewer metastases (ideally 1–3) located in the bone (preferably the spine, or ganglions [45]) and take the patient’s overall condition (e.g. age/Karnofsky score etc.) into account. Oligo-recurrences in other metastatic locations such as in the lung or liver (i.e. without bone or lymphatic involvement) are currently anecdotal in the literature.

### Treatment of bone and lymph node oligo-recurrence in prostate cancer: lessons from palliative treatments and surgery

Surgical experience of prostate cancer bone metastases mainly focusses on spinal metastases, usually as a palliative treatment. Different grading systems or "scores" have been proposed to establish the indication and to select patients suitable for spinal surgery. The first relevant system was published by Tomita [46] and used prognostic factors such as tumor growth/histology, the presence or absence of visceral metastases, and the number of bone metastases to indicate different surgical techniques. These "scores" were evolved and completed by Tokuhashi [47] by taking the patient’s Karnofsky index, the number of vertebral segments affected, and the presence or absence of motor or clinical symptoms into account and is especially reliable for certain prostate and breast metastasis subgroups [48].

Many factors must be considered when selecting different treatments for palliative-care patients including the presence or absence of spinal cord compression syndrome, spinal stability, and histology [49,50]. However, the results from several reports (the majority retrospective) are contradictory and do not show a clear advantage for any particular prognostic sub-group, only a limited benefit of surgery in patients older than 70 years with a radioresistant histology [51-53] . In order to protocolize management and individualize treatment, different algorithms have emerged, for example NOMS, used at the Memorial Sloan Kettering Cancer Center in New York [54]. This system indicates different types of treatment, depending on the patient's clinical neurological and oncological situation, the degree of mechanical spinal instability, and the status of their systemic disease.

Following on from the good results from the phase II Radiation Therapy Oncology Group (RTOG) 0631 trial which assessed the feasibility and safety of spinal SRS, the phase III trial may obtain level 1 evidence for this type of treatment [55] by comparing spinal SRS at a single doses of 16 Gy to conventional treatment at a single dose of 8 Gy in patients with 1–3 spinal metastases. Taken together, these experiences, which are mostly based on palliative-care patients, provide strong evidence that experts should recommend SBRT or SRS rather than conventional external beam radiation in oligometastatic cases [56]. In fact, some RPAs (recursive partitioning analysis indexes) [57] have already been published for spinal radiosurgery.

There are more than 133 clinical trials exploring the role of ablative radiotherapy in different locations, 53 of them in oligometastases and almost 18 in spinal metastases. Focusing on prostate cancer treatment (discussed in greater detail in sections below) current experience suggests that single high doses may be preferable to SBRT or SRS. For example, Muacevic et al. analyzed 40 patients with prostate cancer bone metastases (both spinal and non-spinal); 75% of them were in a single location, and the mean tumor volume was 13 cc. Fifty-four consecutive radiosurgery sessions were administered, with an average single dose of 20.2 Gy and after a mean follow up of 14 months the actuarial local control was 95.5% [58].

In terms of surgical salvage of prostate cancer lymph node metastases, current experience is much more limited compared to other histologies. Randomized studies like that of Verhagen et al. show that combined local surgical treatment and hormone therapy is beneficial in aggressive prostate cancer patients with nodal disease, compared with hormone-only therapy [59]. Recent evidence, as reflected by Haffner et al., shows that lymph node metastases resected during radical prostatectomy did not harbor PTEN, SPOP, TP53, or ATRX mutations, suggesting that these lesions had an independent clonal/sub-clonal origin [60]. No clear surgical benefit was shown, and there was biochemical control in 9-19% of patients at 5 years. Therefore different authors suggest that in order to optimize treatments there must be accurate patient pre-selection [61-63]; indeed, studies using this strategy, such as that of Karnes et al., reach up to 48% five-year progression-free survival (PFS) [64], which has led to efforts to create new algorithms that can select patient subgroups that are most likely to benefit from rescue surgery [65].

Ablative radiotherapy studies of lymph node metastases, both for prostate cancer and other histologies, are still emerging. The work of Bignardi et al., among others, which used SBRT to treat lymph node metastases with various histologies, has shown this treatment to be a safe and effective option and have laid the groundwork for the future study of prostate cancer lymph node metastasis [66-68].

### Difficulties of ablative oligometastases treatment with radiotherapy

Historically there have been a number of difficulties in treating metastases with ablative radiotherapy doses: one is the dose-limit tolerance of healthy tissue to fractionation, a problem that was already successfully solved in the development of cranial radiosurgery [69]. The association of high precision systems for determining the position of the treatment volume using a stereotactic frame, and the subsequent implementation of CT, MRI, and other imaging techniques such as PET/CT allows pinpoint accuracy and ensures adequate dose deposition. The concept of hypofractionation has also been added, which allows for extrapolation at an extracranial level, as well as other modern techniques such as image-guided radiation therapy; together these types of ablative treatments are becoming generalized and now form part of the treatments used in a large number of radiation oncology services.

Another difficulty is identification of patients that truly fit the criteria for oligometastatic disease rather than, for example polymetastatic or oligo-recurrent disease. In spite of this, the development of new imaging techniques such as WB-MRI and the introduction of new PET radiotracers with enhanced diffusability (especially choline) has greatly improved our ability to adequately select patients.

However, the greatest difficulty, and one that is inherent to all ablative treatments, is that different cohorts of oligometastatic sub-types must be taken into account. A patient who is oligometastatic on diagnosis is not the same as one who is oligometastatic after cytoreductive (oligoprogressive) curative locoregional treatment, as they would probably have different prognoses which would require different therapeutic approaches. Therefore it is essential to define the true oligometastatic stage, and as such various theories have been proposed.

Oligometastasis is generally thought of as a situation in which there is a long interval between primary disease treatment and the occurrence of a metastasis, which would have a high percentage growth rate compared to the primary tumor. Indeed, as Withers and Lee [70] state, the greater the doubling time between excision of the primary lesion and clinical detection of a "leader" metastasis in relapsed patients is, the higher the likelihood that the patient has an oligometastatic distribution. Recently, Lussier et al. [71], studied different samples of resected lung metastases in patients with limited metastatic disease, and classified them according to the patient’s rate of disease progression, identifying different microRNA expression patterns in patients with low or high metastatic progression rates. The ability of these phenotypes to predict survival progression was validated, and they showed that microRNAs were able to predict phenotypes in both data sets, differentiating between those who eventually became oligometastatic and those who developed polymetastatic disease. These results support the hypothesis that the presence or development of oligometastases is a clinical entity with distinct biological mechanisms that may differ from those in polymetastatic disease [72].

Thus we can formulate the following hypothesis: patient subgroups with a true oligometastatic disease phenotype can benefit from high-dose ablative therapy which could change their prognosis, and therefore they can receive treatment beyond merely palliative care.

### Fundamentals of bone, spinal and nodal stereotactic body radiation therapy

The foundations of these new techniques can be defined based on three principles: high-dose, high-precision, and high conformation of the dose distribution to the target volume.

#### High radiation doses per fraction: stereotactic ablative radiotherapy

Several reports, such as those by Greco et al. [73] have demonstrated that a high level of local metastasis control (around 90%) can be obtained regardless of the patient’s histology when single fractions over 21 Gy or fractionated doses over 8 Gy are administered. This type of response is due to the radiobiological effect of high doses [74]; while tumor stem cells are the target in conventional radiotherapy, a dose per fraction above 8 Gy causes damage to tumor vessel endothelia, causing intracellular release of ceramides which can also damage to tumor stroma. The same mechanism has been linked to the systemic immunomodulatory effect of these treatments, [75] also defined as the abscopal effect.

Cell apoptosis can be initiated by DNA damage induced by stereotactic ablative radiotherapy (SABR) which causes positive p53 tumor suppressor gene regulation, and may also trigger lipid cell membrane damage. This damage is capable of inducing the formation of ceramides which activate the stress-activated protein kinase/c-Jun N-terminal kinase (SAPK/JNK) signaling pathway which in turn can upregulate protein kinase R (PKR) expression, potentially inducing major histocompatibility complex (MHC) and cytokine expression via NF-kB, resulting in cellular expression of MHC class I, adhesion molecules, co-stimulatory molecules, heat shock proteins, inflammatory mediators, immunomodulatory cytokines, and cell death receptors [76]. This type of effect is being explored with immunotherapy in histologies such as melanoma, and more recently, in prostate cancer [77].

#### High conformal radiotherapy

Precise conformation treatment is required in the use of SABR because there is a high dose gradient between the target volume and the dose-limiting organs at risk. Ablative radiotherapy techniques require precise treatment conformation to maintain correct patient immobilization, accomplished by using one of several commercial immobilization systems which promote patient stability and comfort. It is also very important that these systems are compatible with MRI and PET/CT, given that they may be used for fusion imaging with the radiotherapy planning CT to generate the treatment position. Thus, most radiotherapy planning systems allow either rigid or deformable registration image fusion with other studies. There are currently two trends for ablative vertebral treatments: the use either of fusion imaging with MRI to delineate the spinal cord or a myelogram during the planning CT [78]. In contrast, most studies use choline-PET/CT for nodal metastases.

Another key aspect of conformational high-dose radiotherapy is the development of intensity modulated radiotherapy (IMRT), and more recently, volumetric radiotherapy or arc radiotherapy (VMAT and Rapid Arc) [79] which allow high conformation of the treatment. For the theoretical conformation plotted by the treatment planning system to be successfully translated into a real patient treatment, it must remain stable during the administration and, more importantly, we must ensure that the dose is deposited in the correct place, leading us to the third principle: high precision.

#### High precision

**Figure 1:**
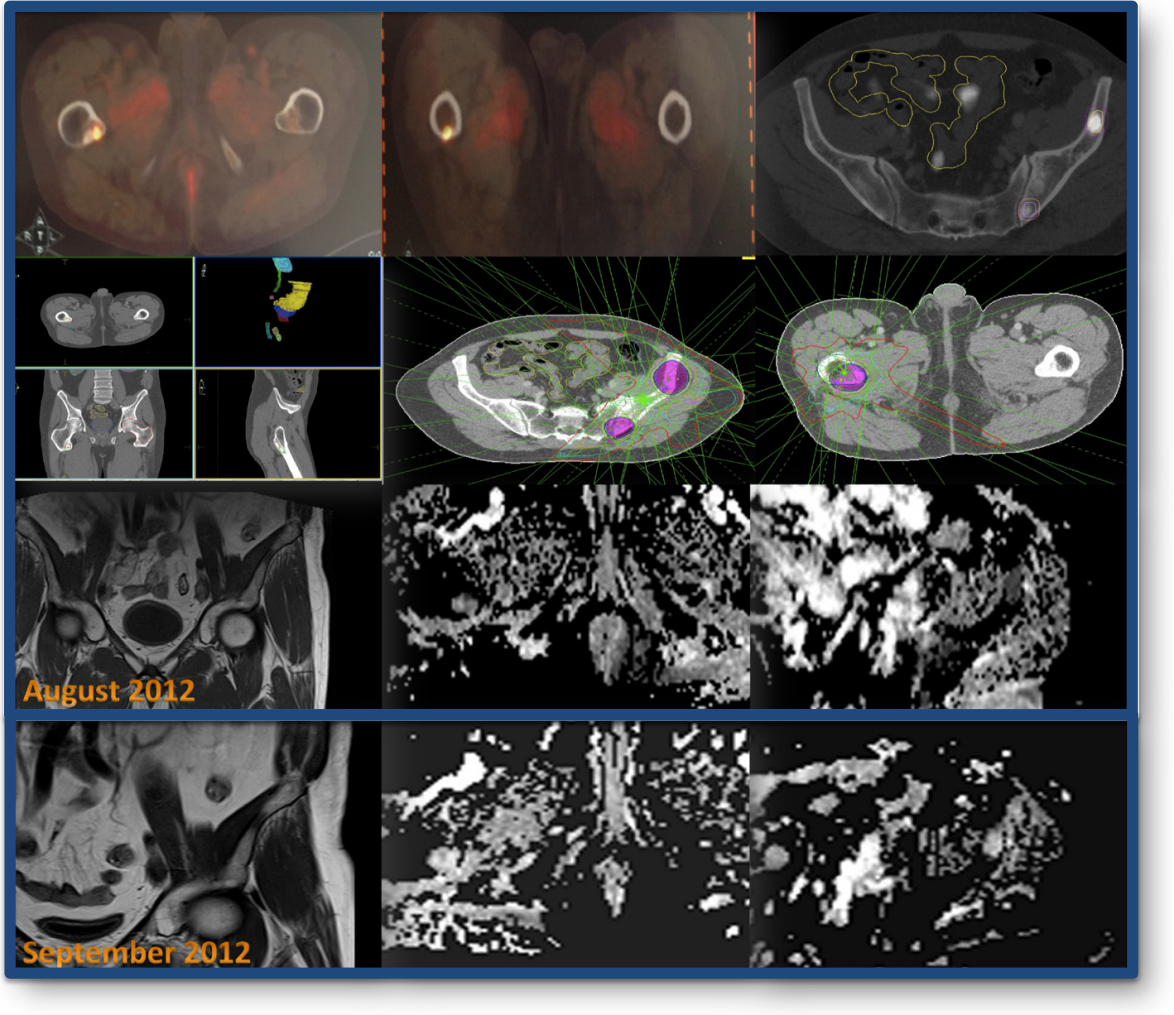
**Case report**: **Patient treated with SBRT and evaluation of response to the changing pattern of ADC in MRI.** Patient diagnosed of 3 bone metastasesof prostate cancer by PET/CT Fluror-Choline and diffusion MRI treated with SBRT (3 fractions of 10 Gy). Assessment by MRI diffusion technique, suggestiveof response to treatment.

The fundamental tool used to give high precision in radiotherapy treatments is the use of image guided radiation therapy (IGRT) [80] which determines the position of the patient, the target volume, and the organs at risk at the time of treatment administration. There are several devices on the market (cone beam tomography with kilovoltage, megavoltage, stereoscopic images, infrared sensors, laser surface scanning systems, or by implantable radiofrequency position sensors), which can all be combined and integrated in different ways in different commercial solutions Figure 1.

### Current experience in radiotherapy treatment of prostate cancer oligometastases

There are several documented experiences that focus on radiation treatment of prostate cancer oligometastases and although most of them focus on general oligometastasis treatment or on spinal or nodal metastases, some prostatic-origin cases are included because it is a common presentation [81].

Using 3D dynamic IMRT techniques, Schick et al. [82] published data on 22 oligometastatic prostate cancer patients; 55% of them presented only one lesion and received combined treatment with hormones and doses up to 65 Gy with conventional fractionation. 18F-choline or 11C-acetate PET-CT were used to diagnose and delineate the lesions, and identified 9 cases of local nodal relapse, 6 of distal nodal relapse, 6 bone metastases, and 1 visceral metastasis. With a median follow-up of 39 months (range 11–75 months), biochemical relapse-free survival (BRFS) at 3 years was 63% and OS was 89%. Multivariate analysis established that BRFS is a variable associated with an age over 61 years.

Another study using IMRT techniques and conventional fractionation, Würschmidt et al. [83] analyzed 26 patients, 7 of them primary tumors, and 19 recurrences. They received a median dose of 75.6 Gy to the primary tumor and 66.6 Gy to lymph node sites. OS at 28 months was 94% and BRFS was 83% for the primary tumor and 49% for recurrences and 100% for distant disease-free survival in the primary tumor and 75% in recurrences.

Focusing on ablative radiotherapy schedules in patients with a local relapse, Casamassina et al. [84] analyzed data from 71 patients who had had different primary treatments (post-prostatectomy, post-radiotherapy, or both), all of whom were treated using SBRT techniques. Thirteen patients presented persistent disease regression, two had bone metastases, and 8 had lymph node recurrences (all of them outside the irradiated areas). PSA fell from 5.65 ng/ml to a median 1.40 ng/ml in complete regression patients, and the nadir value (median 1.06 ng/ml) was maintained for a median of 5.6 months. Using a Cyberknife® Jereczek-Fossa et al. [85,86] reported data from 38 lesions in 34 patients; 15 of them were re-irradiated after local relapse, four after an anastomosis relapse, 16 after nodal relapse, and 3 were treated for a single metastasis (two retroperitoneal and one bone). PET/CT with 11C-choline was used for diagnosis in 30 cases and a 33 Gy 3-fraction or 36 Gy 3-fraction dose scheme was used to treat lymph node or bone metastases respectively.

Median follow-up was 16.9 months. Acute urinary toxicity was described in 8 patients (in three cases grade 1 and in five cases grade 2) and rectal toxicity presented only in one case (grade 1). Late-onset urinary toxicity occurred in five cases (in three cases grade 3 and in two cases grade 2) and one case each of grade 1 and grade 2 late-onset rectal toxicity was described. A biochemical response was observed in 32 of 38 evaluable lesions, PSA stabilization was observed in four lesions, and in two cases PSA progression was reported. PFS was 42.6% at 30 months and there was disease progression in 14 lesions (5, 2, 5, and 2 cases in the prostate, anastomosis, lymph node, and metastasis groups respectively); progression was observed in only three cases. At the time of analysis, 19 patients were alive without evidence of disease and 15 were alive with disease. In the case of nodal metastases, at a median follow-up of 21.9 months 16 cases presented solely nodal relapse, 10 had a complete response, there was one case of stable disease, and five cases of disease progression, as assessed by choline-PET/CT. At the level of PSA response, 12 complete responses, one partial response, two cases of stable disease, and one case of disease progression were reported. With regard to the three metastases, at a median follow-up of 13.7 months, there was one case of radiological progression outside the treatment field, one case of disease progression due to a rise in PSA, and one complete response. The median PFS time was 11 months (range: 6–16 m) and no cases of toxicity were reported.

Using single doses of SRS with a Cyberknife® Muacevic et al.[59] published data from a series of 40 patients; of these, 19 received hormone therapy, and 64 bone metastases were detected using 18F-choline PET/CT which were treated with a mean single dose of 20.2 Gy (range: 16.6-22 Gy). Seventy-five percent of patients had only one bone metastasis and 25% had two. Only 8 patients received chemotherapy prior to ablative radiotherapy. At a median follow-up of 14 months (range: 3–48 m) 95.5% local control was described, as assessed by MRI and PET/CT. Prior to SRS the median PSA was 5.4 ng/dl which decreased to 2.7 ng/dl at three months. One patient developed progressive neurological deficits after treatment.

With respect to the management of oligometastatic patients using ablative radiotherapy to delay the onset of hormone therapy, Berkovic et al. [87] published their experience in the treatment of 24 patients with a biochemical relapse, after initial treatment for three or fewer metastases detected using choline-PET/CT, who had not received hormone therapy. These patients were treated with a schedule of 50 Gy in 10 fractions. Where biochemical control was not achieved or new metastases were detected (total fewer than three) during monitoring, radiation therapy was re-administered. The authors report 100% two-year local control, and 42% PFS at 2 years. Patients started hormone therapy if PSA levels were greater than 50 or if the number of metastases was greater than three; ten patients ultimately received this treatment and had a median androgen deprivation therapy-free survival of 38 months. No cases of grade 3 toxicity were described. This is the first work to show that treatment with ablative therapy can delay the use of systemic therapies and has led to a phase II trial headed by Dr. Piet Ost at the Ghent University Hospital (currently recruiting) comparing surveillance versus SBRT treatment or surgery in patients with prostate cancer oligometastases, with the measure of androgen deprivation-free survival as primary endpoint [88].

Recently, Picchio et al. [45] published the results from 83 patients with lymph node relapses after radical primary treatment with simultaneous integrated boost (SIB) therapy combined with Tomotherapy. Sixty-six patients experienced a complete biochemical response, 12 a partial biochemical response, 15 had disease progression, and one had stable disease. Ahmed et al. also described 100% local control with SBRT and an undetectable PSA nadir in nine (53%) of 17 patients (with 21 lesions), 65% of whom had hormone-refractory disease [89].

In summary, the major schemes used for treating prostate cancer oligometastases are all based on SBRT, with an average dose range of one fraction of 20 Gy to three fractions of 10 Gy. Other schemes used are 5–6 fractions of 5 or 6 Gy and 10 fractions of 5 Gy, with no discernable difference between the two. Concepts such as the logistics of radiation oncology services and the possible advantage of hypofractionation doses versus high single doses should be considered in future studies [90] (Table 1). Hormone deprivation, as reflected in the 2013 EAU recommendations, [91], clearly delays clinical progression and prolongs survival in patients with asymptomatic prostate cancer. Therefore, a phase II clinical trial (currently recruiting) [92] led by Conde et al. at the *Hospital Provincial de Castellón* (Spain) and sponsored by the Spanish research group in radiation oncology (GICOR), the Spanish Society of Radiation Oncology (SEOR), and the Spanish SBRT group (SBRT-SG) is seeking to uncover any association between hormone therapy and SBRT for these oligometastases and whether these treatments can increase local control, PFS, and chemotherapy-free survival. Another phase II prospective clinical trial from the University of Florida is exploring the efficacy and safety of SBRT in patients with metastatic prostate cancer, either with or without an active primary tumor, in two subgroups: castration-resistant and hormone-receptive patients [93]. Other clinical trials include the treatment of prostate oligometastases, but do not focus only in this histology [94] (Table 2).

**Table 1:** **Published studies in the literature about ablative and radical radiotherapy in the management of oligometastases**

**Author**	**Year of publication**	**Type of lesions**	**Treatment received**	**Results**
*Casamassine et al* [84]*.*	2011	71 patients:	No ADT	- 13 persistent regression
		- 28 post-prostatectomy		
		- 15 post-radiotherapy		- 2 bone metastases
		- 28 post-prostatectomy and radiotherapy		- 8 lymph node recurrences (outside the irradiated áreas)
*Muacevic et al.* [58]*.*	2011	64 bone metastases	-19 ADT	95% local control
			- Mean dose 20,2 Gy (range 16,6-22 Gy)	
			- 8 patients chemotherapy	
Würschmidt *et al.* [83]*.*	2011	26 patients	Mean dose 75,6 Gy (primary site) and 66,6 Gy (lymph node sites)	- Overall survival at 28 m: 94%
				- Biochemical relapse free survival (primary site): 83%; 49% (recurrences)
				- Distant free survival 100% (primary site) and 75% (recurrent)
*Ahmed et al.* [89].	2013	17 patients (21 lesions)	Mean dose 20 Gy in 1 to 3 fractions	- Local control rates 100%
				- 2-5 year progresión-free survival 20%
*Berkovic et al.* [87]*.*	2012	24 patients with biochemical relapse after initial treatment	- SBRT 50 Gy in 10 fractions	- 100% 2-year local control
			- None ADT	
				-Progr. free survival at 2 years 42%
				- ADT: median survival free 38 months
*Jereczek-Fossa et al.* [85]*.*	2012	34 re-irradiated patients: 15 local relapse, 4 anastomosis, 16 nodal and	23 Gy in 3 fractions in lymph node metastases and 36 Gy in 3 fractions in bone.	- 32 biochemical response
				- 4 PSA stabilization
		3 distant metastases (2 retroperitoneal, 1 bone)		- 2 PSA progression
				- 17 disease progression
				- Progression free survival at 30 m: 42,6%.
*Shick et al.* [82]*.*	2013	22 oligometastasic patients (55% one lesión)	55% ADT + EBRT (65 Gy)	- Biochemical relapse-free survival at 3 y: 63%
				- Overall survival 89%
*Picchio et al.* [45]*.*	2014	83 patients biochemical recurrence after radical primary treatment	No ADT	- 66 patients complete biochemical response
				- 12 partial biochemical response
				- 1 stable disease
				- 15 progression disease

**Table 2:** **Current ongoing trials for prostate cancer oligometastases in 2014** (**www.clinicaltrials.gov**)

**Study**	**ClinicalTrials.gov identifier**	**Phase**	**Aim**	**Arms**	**Primary objetives**	**Secondary objetives**
**Radiotherapy for oligometastatic prostate cancer**	NCT01859221	2	Efficacy and safety in patients with prymary active or not	2: CR and HN	Improvement in median progression-free survival in patients with metastatic prostate cancer over historic control rates in hormone receptive and castration resistant subgroups.	Improvement in overall survival of patients with metastatic prostate cancer.
University of Florida						
						Treatment failure rates in patients treated with stereotactic radiation for metastatic prostate cancer. after type of secondary outcome.
						Quality of life in patients treated with stereotactic radiation for metastatic prostate cancer.
						(CTCAE 4.0) adverse events other than a dose-limiting toxicity which is possibly, probably, or definitely related to treatment and which occurs within 6 months from the start of SBRT to multiple metastases.
						II. To estimate the rates of long-term adverse events occurring up to 2 years from the end of SBRT.
						III. To explore the most appropriate and clinically relevant technological parameters to ensure quality and effectiveness throughout radiation therapy processes, including imaging, simulation, patient immobilization, target and critical
						structure definition, treatment planning, image guidance and delivery.
**Stereotactic Radiosurgery in Treating Patients With Metastatic Breast Cancer,** **Non-** **Small Cell Lung Cancer,** **or Prostate Cancer**	NCT02206334	1	Safety Study	1	To determine the recommended SBRT dose for each of the metastatic locations being treated given the individual and overlapping fields when multiple metastases are treated with SBRT in a national clinical trials network setting.	I. To estimate rates of > = grade 3
NRG Oncology Foundation, Inc.						
Collaborator:						
NCI RTOG						
**Non-** **systemic Treatment for Patients With Low-** **volume Metastatic Prostate Cancer**	NCT01558427	2	Defer the start of ADT	2: A. Active surveillance	Androgen deprivation therapy free survival.	Quality of life
				B. Surgical or radiotherapy treatment of metastases		
University Hospital, Ghent						
**Phase II Study of SBRT as Treatment for Oligometastases in Prostate Cancer**	NCT02192788	2	Safety and Efficacy Study	1	Local and symptomatic control of oligometastases treated by SBRT	Biochemical progression rates
Consorcio Hospitalario Provincial de						
Castellón						
Collaborators:						Progression-free survival,
SBRT-SG						
						Chemotherapy-free survival and overall survival.
GICOR						
						Analyze toxicities and quality of life of patients before and after treatment
SEOR						

CR: Castrate resistant HR: Hormone Receptive NCI: National Cancer Institut RTOG: Radiation Therapy Oncology Group ADT: androgen deprivation therapy.

CTCAE 4.0: Common Terminology Criteria for Adverse Events SBRT-SG: Sterotactic Body Radiation Therapy Spanish Group.

GICOR: Spanish Group of clinical Investigation in Radiation Oncology SEOR: Spanish society of Radiation Oncology.

In terms of the general role that ablative radiotherapy has in the management of oligometastases, Tree et al [95] published a review based on evidence and expert recommendations. This type of treatment allows a high degree of treated-metastasis control (around 80%) to be gained with low toxicity, with 20% of patients being progression-free two to three years after treatment. The patients who had longer disease-free intervals were those with more radiosensitive histologies, followed by those with pulmonary or colorectal origin metastases, fewer metastases (1–3), and where high-dose radiation treatments were administered with a biologically effective dose higher than 100 Gy. Therefore these types of treatments are a good option to consider for patients with isolated metastases (fewer than 3–5 lesions) and a previous disease-free interval of more than 6 m. These facts underscore the need for more randomized trials in order to establish whether PFS and OS increase when ablative radiotherapy treatments are implemented, as well as to compare them to surgery.

Undoubtedly, the two greatest benefits that have come from all these types of therapies are the development of high quality imaging for detecting metastases, and the knowledge of new molecular markers (such as microRNAs) and their expression patterns, meaning that tumor biopsies can be used to identify poor-prognosis oligometastatic patient subgroups who may benefit from systemic treatments, before or after the discovery of new metastases. Furthermore, future ablative radiotherapy treatments may also be administered in combination with bisphosphonates and denosumab as well as hormone therapy. Moreover, it are expected to the new anti-androgens (such as abiraterone and enzalutamide) and target therapy drugs may promote greater control of systemic disease by acting on micrometastases while radiation acts on macroscopic disease [96,97] (“Search strategy and selection criteria”).

### Search strategy and selection criteria

Studies were identified in July, 2014, by searches of PubMed, www.clinicaltrials.gov and Web of Knowledge (search terms included combinations of oligometastases, prostate, stereotactic, radiotherapy, metastases, radiosurgery, SBRT, and SABR), and by review of cited papers in selected articles. We identified 435 papers. We assessed retrospective and prospective studies published in English. We excluded duplicates or studies reporting a mixture of hystologies, or non oligometastatic disease. Studies were eligible if the dose per fraction was 6 Gy or higher for a total dose of more than 24 Gy, or 5 Gy per fraction for a total dose of more than 45 Gy. Studies were excluded if fewer than seven patients were reported or if median follow-up was less than 12 months. 8 studies in oligometastatic prostate cancer met the eligibility criteria for this Review, and 5 focused in SBRT/SRS.

## Conclusions

The main conclusions that can be drawn from the evidence presented here can be summarized in the following points:

1. More clinical and molecular markers which can classify patients with stage IV cancers are becoming available.
2. Different approaches must be taken for patients with polymetastatic, oligometastatic, or oligo-recurrent disease because these pathologies are different entities.
3. SBRT and SRS are now alternative treatment options for oligometastatic patients with various diseases such as colorectal or lung cancer, and there is evidence from different studies that suggests that these treatments may also be a viable alternative for prostate cancer patients.

Several prospective ongoing trials may help to clarify the role of ablative therapy in the face of increasing hormone-free and chemotherapy-free survival rates in oligo-recurrent prostate cancer patients.

## Abbreviations

SBRT: Stereotactic body radiation therapy
SRS: Stereotactic radiosurgery
PSA: Prostatic specific antigen
CT: Computed tomography
MRI: Magnetic resonance imaging
WB: Whole-body
DWI: Diffusion-weighted imaging
PET: Positron emission tomography
NCCN: National Comprehensive Cancer Network
OS: Overall survival
PCSS: Prostate cancer-specific survival
PSA-DT: Pre-metastatic PSA duplication time
RTOG: Radiation Therapy Oncology Group
RPAs: Recursive partitioning analysis index
PFS: Progression-free survival
SABR: Stereotactic ablative radiotherapy
SAPK/JNK: Stress-activated protein kinase/c-Jun N-terminal kinase
PKR: Protein kinase R
MHC: Major histocompatibility complex
IMRT: Intensity modulated radiotherapy
VMAT: Volumetric radiotherapy
IGRT: Image Guided Radiation Therapy
BRFS: Biochemical relapse-free survival
SIB: Simultaneous integrated boost
GICOR: Spanish research group in radiation oncology
SEOR: Spanish Society of Radiation Oncology
SBRT-SG: Spanish SBRT group
