# Erratum: Oligometastases in prostate cancer: restaging stage IV cancers and new radiotherapy options

**DOI:** 10.1186/s13014-015-0444-2

**Published:** 2015-07-28

**Authors:** Antonio José Conde Moreno, Carlos Ferrer Albiach, Rodrigo Muelas Soria, Verónica González Vidal, Raquel García Gómez, María Albert Antequera

**Affiliations:** Servicio de Oncología Radioterápica, Instituto Oncológico de Castellón “Dr. Altava”, Consorcio Hospitalario Provincial de Castellón, Av. Dr. Clarà N 19, Castellón de la Plana, 12002 Castellón Spain

## Erratum

After the publication of this work [1], we noticed that an incorrect version of Table two (Table 1 here) [1] was published. The correct version of Table two (Table 1 here) is provided here.

**Table 1:** Current Ongoing trials for Prostate Cancer Oligometastases in 2014 (www.clinicaltrials.gov)

Study	ClinicalTrials.gov Identifier	Phase	Aim	Arms	Primary Objetives	Secondary Objetives
Radiotherapy for Oligometastatic Prostate Cancer	NCT01859221	2	Efficacy and safety in patients with prymary active or not	2: CR and HN	Improvement in median progression-free survival in patients with metastatic prostate cancer over historic control rates in hormone receptive and castration resistant subgroups.	Improvement in overall survival of patients with metastatic prostate cancer.
University of Florida						Treatment failure rates in patients treated with stereotactic radiation for metastatic prostate cancer. after type of secondary outcome.
						Quality of life in patients treated with stereotactic radiation for metastatic prostate cancer.
Stereotactic Radiosurgery in Treating Patients With Metastatic Breast Cancer, Non-Small Cell Lung Cancer, or Prostate Cancer	NCT02206334	1	Safety Study	1	To determine the recommended SBRT dose for each of the metastatic locations being treated given the individual and overlapping fields when multiple metastases are treated with SBRT in a national clinical trials network setting.	I. To estimate rates of > = grade 3 (CTCAE 4.0) adverse events other than a dose-limiting toxicity which is possibly, probably, or definitely related to treatment and which occurs within 6 months from the start of SBRT to multiple metastases.
NRG Oncology Foundation, Inc.						II. To estimate the rates of long-term adverse events occurring up to 2 years from the end of SBRT.
Collaborator: NCI RTOG						III. To explore the most appropriate and clinically relevant technological parameters to ensure quality and effectiveness throughout radiation therapy processes, including imaging, simulation, patient immobilization, target and critical structure definition, treatment planning, image guidance and delivery.
Non-systemic Treatment for Patients With Low-volume Metastatic Prostate Cancer	NCT01558427	2	Defer the start of ADT	2: A. Active surveillance	Androgen deprivation therapy free survival.	Quality of life
University Hospital, Ghent				B. Surgical or radiotherapy treatment of metastases		
Phase II Study of SBRT as Treatment for Oligometastases in Prostate Cancer	NCT02192788	2	Safety and Efficacy Study	1	Local and symptomatic control of oligometastases treated by SBRT	Biochemical progression rates
GICOR						Progression-free survival,
Collaborators:						Chemotherapy-free survival and overall survival.
SBRT-SG						Analyze toxicities and quality of life of patients before and after treatment
SEOR						
Consorcio Hospitalario Provincial de Castellón						

*CR* Castrate resistant, *HR* Hormone Receptive, *NCI* National Cancer Institut, *RTOG* Radiation Therapy Oncology Group, *ADT* androgen deprivation therapy

CTCAE 4.0: Common Terminology Criteria for Adverse Events SBRT-SG: Sterotactic Body Radiation Therapy Spanish Group

GICOR: Spanish Group of clinical Investigation in Radiation Oncology SEOR: Spanish society of Radiation Oncology
